# Biventricular interaction and aortic function in adult patients with repaired tetralogy of Fallot: a two-dimensional–three-dimensional speckle-tracking echocardiographic study

**DOI:** 10.1093/ehjimp/qyae015

**Published:** 2024-03-02

**Authors:** Antonio Vitarelli, Lidia Capotosto, Fabio Miraldi, Khaled Mukred, Marco Francone, Nicola Galea, Enrico Mangieri, Gaetano Tanzilli, Nicola Viceconte, Massimo Mancone, Bich Lien Nguyen, Costantino Smaldone, Sulaiman Al-Kindy

**Affiliations:** 1 Cardiodiagnostica CS, Via Lima 35, Rome 00198, Italy; Department of Cardiology, Cardiac Surgery and Radiology, Sapienza University, Rome, Italy; 1 Cardiodiagnostica CS, Via Lima 35, Rome 00198, Italy; Department of Cardiology, Cardiac Surgery and Radiology, Sapienza University, Rome, Italy; Department of Cardiology, Cardiac Surgery and Radiology, Sapienza University, Rome, Italy; University Teaching Hospital, Department of Cardiology and Medicine, Dhamar, Yemen; Department of Cardiology, Cardiac Surgery and Radiology, Sapienza University, Rome, Italy; Department of Cardiology, Cardiac Surgery and Radiology, Sapienza University, Rome, Italy; Department of Cardiology, Cardiac Surgery and Radiology, Sapienza University, Rome, Italy; Department of Cardiology, Cardiac Surgery and Radiology, Sapienza University, Rome, Italy; Department of Cardiology, Cardiac Surgery and Radiology, Sapienza University, Rome, Italy; Department of Cardiology, Cardiac Surgery and Radiology, Sapienza University, Rome, Italy; Department of Cardiology, Cardiac Surgery and Radiology, Sapienza University, Rome, Italy; Department of Cardiology and Cardiac Surgery, San Carlo Hospital, Potenza, Italy; Department of Cardiology, Cardiac Surgery and Radiology, Sapienza University, Rome, Italy

**Keywords:** right ventricular function, left ventricular function, three-dimensional speckle-tracking echocardiography, aortic function, repaired tetralogy of Fallot

## Abstract

**Aims:**

In patients late after correction of tetralogy of Fallot (TOF), the combined effects of pre-operative hypertrophy and hypoxia, ventricular interdependence, acquired post-operative lesions such as pulmonary or aortic regurgitation, and congenital vasculopathy may result in impaired right ventricular (RV) and left ventricular (LV) function. The aim of the present study was to investigate the interventricular interactions in repaired TOF (rTOF) and the impact of aortic function on biventricular performance using two-dimensional (2D-STE) and three-dimensional speckle-tracking echocardiography (3D-STE).

**Methods and results:**

Twenty-five adult patients with rTOF and 25 age- and gender-matched healthy controls were studied. LV and RV volumes were determined by 3D-STE and cardiac magnetic resonance. LV and RV longitudinal strains (LVLS and RVLS) and LV and RV area strains (LVAS and RVAS) and LV twist/rotation were calculated by 3D-STE. Ascending aorta circumferential strain (AAo-CS) was obtained using 2D-STE. LV 3D-STE parameters were decreased in rTOF patients compared with controls even in patients with normal ejection fraction. AAo-CS was decreased (6.7 ± 1.9 vs. 10.1 ± 2.6, *P* = 0.003) in rTOF patients compared with controls even in the presence of normal aortic dimensions and correlated with AAo diameter (*r* = −0.69, *P* = 0.0001), LV twist (*r* = 0.54, *P* = 0.004), LVAS (*r* = −0.56, *P* = 0.003), and RVLS (*r* = −0.39, *P* = 0.036). LVAS and AAo-CS were associated with disease severity (peak oxygen consumption and arrhythmia occurrence). Significant improvement in global *χ*^2^ value was noted with RV 3D-STE parameters + LVAS + AAo-CS compared with RV dysfunction alone for detecting exercise capacity impairment (from 77.1 to 84.4 to 91.2, *P* = 0.003).

**Conclusion:**

Speckle-tracking echocardiography revealed subtle LV and AAo dysfunction in adults with rTOF. A correlation was observed between LV and RV strain changes and between AAo strain impairment and LV/RV dysfunction. LV and AAo changes had an incremental value in evaluating disease severity.

## Graphical Abstract

**Graphical Abstract:**
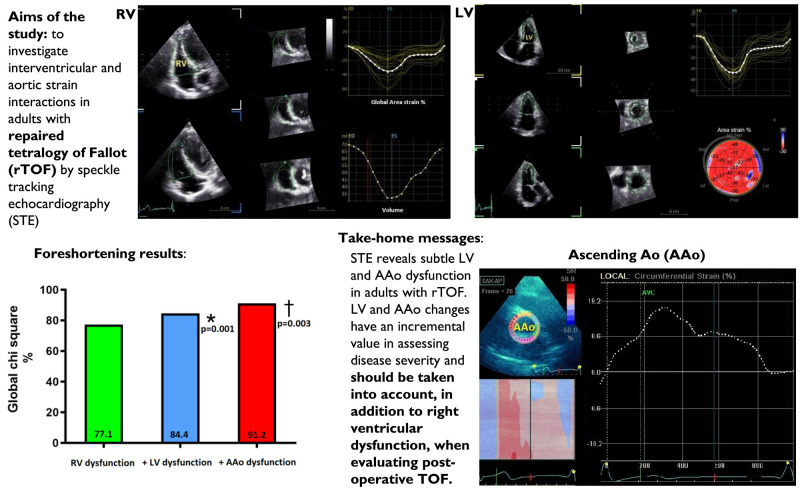

## Introduction

Although early surgical repair of tetralogy of Fallot (TOF) has significantly improved long-term outcomes, risk stratification for survival is still difficult to detect, and some patients remain at high risk for premature cardiovascular death.^1^ Right-sided cardiac chambers have received attention over the years in the management of patients with repaired TOF (rTOF).^2–4^ However, several studies have described a correlation between right ventricular (RV) and left ventricular (LV) systolic and diastolic dysfunction in patients with rTOF suggesting a ventricular interaction.^5,6^ Decreased LV ejection fraction (LVEF) has been reported in about 20% of adult rTOF patients, and subclinical LV dysfunction was present even in patients with normal LVEF.^6^ It was suggested that LV function could be taken into consideration in the timing of pulmonary valve replacement (PVR) as an additional indication^7^ and recent guidelines include a combination of RV volumes and biventricular function to guide PVR timing in these patients.^8^

The pathophysiology of the left heart has generally been attributed to the concurrent impact of right heart dysfunction, but abnormalities of aortic wall function in rTOF have also been shown in recent studies.^9,10^ Although aortic dilation has been associated with volume overload due to haemodynamic alterations in unrepaired TOF, abnormal dimensions and compromised elastic properties may also be found in the ascending aorta in rTOF late after surgical correction.^9^ Histopathological studies confirmed intrinsic aortic wall abnormalities, which may be the cause of elastic abnormalities and progressive aortic dilation.^11^ High wall shear stress has also been demonstrated throughout the proximal aorta in pre-adolescent rTOF patients even in the presence of normal aortic dimensions,^12^ and impaired distensibility of the proximal aorta was observed in fetuses with TOF.^13^

Accordingly, since biventricular and aortic function using two-dimensional (2D-STE) and three-dimensional speckle-tracking echocardiography (3D-STE) has not been comprehensively explored in rTOF, we hypothesized that these techniques could provide new insights into the pathophysiology of this complex disease and ventriculo-aortic inter-relationships. Therefore, the aim of the present study was three-fold: (i) to investigate interventricular interactions in rTOF by 3D-STE, (ii) to assess the impact of 2D-STE-derived aortic strain on biventricular performance, relationship to aortic diameter, and comparison with standard echocardiography, and (iii) to analyse the clinical value of STE parameters.

## Methods

Twenty-five adult patients with rTOF and New York Heart Association (NYHA) Classes 1 and 2 referred to the Echocardiology Unit of the Sapienza University Hospital were included retrospectively. All forms of surgical repair were considered, including cases where a palliative shunt was performed prior to surgical correction. Exclusion criteria were significantly associated cardiac lesions, residual ventricular septal defect, significant RV outflow obstruction (Doppler-derived pressure gradient ≥ 40 mmHg), RV to pulmonary artery conduits, or echocardiographic recordings of inadequate quality. The echocardiographic protocol was applied prospectively for a period from June 2019 to March 2022, and expert cardiologists were blinded to any other results. Cardiac magnetic resonance (CMR) and cardiopulmonary exercise testing (CPET) were performed within 3 days of the echocardiographic study. The clinical research design was approved by the University Department Board, and patient-informed consent was obtained. Twenty-five age- and gender-matched healthy controls comprised a random sample from the general population and were enrolled from subjects followed clinically for non-specific chest pain and palpitations with no detectable organic cause.

### 2D echocardiography

All patients underwent transthoracic echocardiography with a commercially available cardiovascular ultrasound system (Vivid E9 or E95, GE, Horten, Norway). Greyscale recordings were optimized at a mean frame rate of ≥50 frames/s. Measurements of cardiac chambers and aortic diameters were made according to established criteria.^14^ 2D measurements of aortic diameters were made from leading edge to leading edge at end-diastole (QRS complex onset) in parasternal long-axis views at the level of the sinuses of Valsalva, the sinotubular junction, and the proximal ascending aorta (2–3 cm above the sinotubular junction). Aortic size was considered both as an absolute value and relative to the *z*-score. Aortic sinuses and ascending aorta *z*-scores were obtained using standard nomograms for aortic size in healthy adults.^15^ RV systolic pressure was determined by continuous wave Doppler echocardiography.^14^ Peak early (*E*) and late (*A*) diastolic velocities were obtained using standard Doppler practices. Mitral and tricuspid annulus velocities (*S_a_*, *E_a_*, and *A_a_*) were measured by tissue Doppler imaging at the lateral annulus on the transthoracic four-chamber views.

### Biventricular 3D speckle-tracking echocardiography

**Figure 1:**
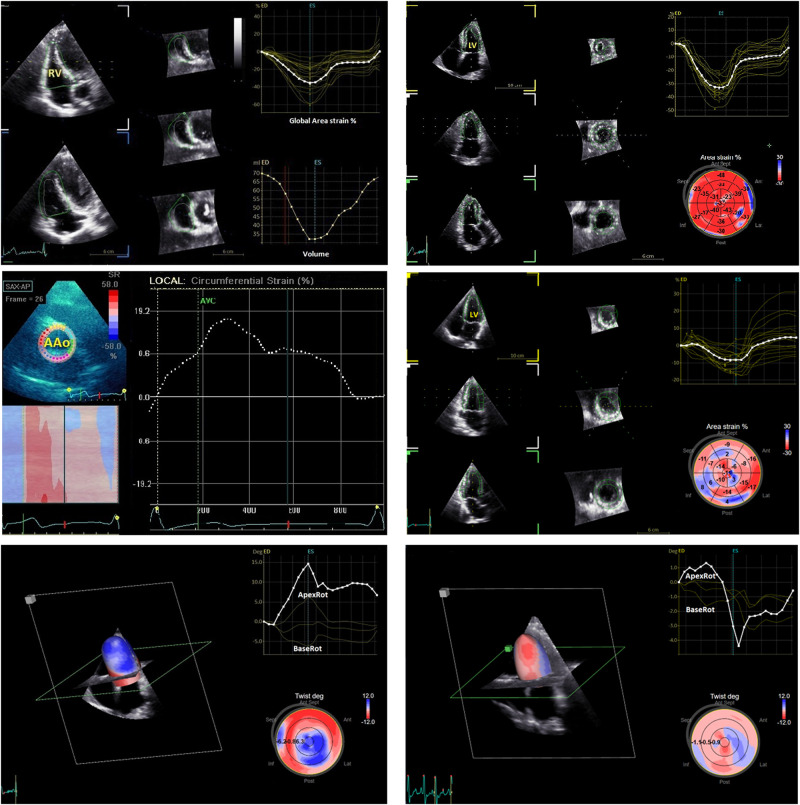
Representative speckle-tracking echocardiography images in normal subjects and rTOF. (Top, left) RV 3D volume–time and strain–time curve analysis in a normal subject. (Top, right) Regional 3D strain curves of LV segments in a normal subject. (Mid, left) Regional strain curves of AAo segments in a normal subject. (Mid, right) Regional 3D strain curves of LV segments in a rTOF patient. There is lower amplitude and more dyssynchrony in the rTOF patient. LV global area strain is −31% in the control subject and −10% in the patient. (Bottom, left) Apical and basal rotation and twist curves in a normal subject. 3D images (online 3D reconstruction and bull’s-eye diagram) show prominent apical rotation (bluish apical changes in LV twist). (Bottom, right) Apical and basal rotation in a rTOF patient, with impairment of twist and apical rotation curves. 3D images (online 3D reconstruction and bull’s-eye diagram) show an impairment of apical rotation pattern (reddish changes in LV twist). 3D, three-dimensional; AAo, ascending aorta; ApexRot, rotation at apical level; BaseRot rotation at basal level; LV, left ventricle; RV, right ventricle.

3D examination was carried out with the patient lying in the left lateral recumbent position, and analysis was performed by using a 4 V phased-array transducer with a high volume rate (≥30 image frames/s). Standard parasternal, apical, and subcostal views were used. 3D images were saved in digital form on the hard disk of the ultrasound scanner and subsequently analysed using the EchoPAC view (Version-R6, GE Vingmed Ultrasound, Horten, Norway). The LV and RV end-diastolic volumes and LV and RV end-systolic volumes were measured from each 3D echocardiographic data set. LVEF and RV ejection fraction (RVEF) were determined. The process of volume determination was done two times for each patient. Papillary muscles were not included in the volume estimation. LV longitudinal strain (LVLS) was calculated in three apical views in relation to the strain value at aortic valve closure and measured in 17 segments based on the software bull’s-eye diagram.^16,17^ Strain values were not derived in the presence of more than two suboptimal segments in a single apical view. Circumferential and radial systolic strains were calculated as an average of strain values obtained from the basal, mid, and apical parasternal short-axis views. LV area strain (LVAS)^16^ was calculated as the percentage decrease in the size of endocardial surface area defined by vectors of longitudinal and circumferential deformation at end-systole from its original area at end-diastole. The time rotation curves were displayed along the cardiac cycle (*Figure 1*). Counterclockwise rotation was conventionally marked as a positive value and clockwise rotation as a negative value when viewed from the LV apex. Peak apical and basal rotation and peak LV twist were obtained (*Figure 1*). LV twist^17^ was defined as the net difference (in degrees) of apical and basal rotation at isochronal time points. LV torsion (LVtor) was normalized twist divided by LV ventricular diastolic longitudinal length between apex and mitral plane. RV longitudinal strain (RVLS) and RV area strain (RVAS)^4,18^ were recorded for the RV myocardial free-wall (RVFWLS) and septal segments and the entire RV wall (RVLS). The global strain was calculated by averaging local strains along the entire RV using machine software (*Figure 1*).

### Aortic function

To assess aortic distensibility echocardiographic tracings were obtained using a 2D guided M-mode evaluation of systolic and diastolic aortic diameter, 3 cm above the aortic valve. The elastic properties of the aorta were indexed by calculation^19–21^ of aortic distensibility (*D*) and stiffness index (SI) as *D* = 2(*A_s_* − *A_d_*)/[*A_d_* (*P_s_* − *P_d_*)], and SI = ln(*P_s_*/*P_d_*)/(*A_s_* − *A_d_*)/*A_d_*, respectively, where *A_s_* is the aortic diameter at end-systole, *A_d_* is the aortic diameter at end-diastole, *P_s_* is the systolic blood pressure, *P_d_* is the diastolic blood pressure, and ln is the natural logarithm. Aortic wall TDI velocities were obtained by marking a region of interest on the 2D image in the anterior aspect of the ascending aorta at the same point as in M-mode measurements. Systolic maximum wall expansion velocity (AoSvel, cm/s) and wall contraction early diastolic velocity (AoEvel, cm/s) were derived.

The 2D-STE technique was used to calculate the ascending aorta circumferential wall strain (AAo-CS).^22^ Images were analysed (GE EchoPac software version-R6) by drawing a line along the inner side of the aortic wall in the short axis, minimizing the width of the region of interest. Additional lines were generated by the software on the outer side of the vascular wall. In case of unsatisfactory tracking of the wall, manual adjustments were made or software parameters such as the smoothing function or the size of the region of interest were changed. The aortic wall was divided into six segments: anterior right, anterior left, left, posterior left, posterior right, and right. The numerical values of each region, representing the average values of all segmental points, were colour coded and plotted as a function of time throughout the cardiac cycle creating quantitative curves (*Figure 1*). This process and the conversion to Lagrangian strain were performed offline using dedicated software. Global AAo-CS was calculated as mean of peak value of six segments and corrected for pulse pressure.^22,23^

### Cardiac magnetic resonance

A 1.5-T unit (Avanto; Siemens, Erlangen, Germany) with an 8-element phased-array coil was used for the CMR exams. Standard protocol enclosed cine balanced steady-state free precession imaging, T2-weighted black-blood short inversion time turbo spin-echo, and T1-weighted contrast-enhanced gradient-echo imaging with inversion recovery. A dedicated software (cvi42 4.1.8, Circle Cardiovascular Imaging Inc, Calgary, Canada) was used for the analysis of the CMR images. Tracing of ventricular endocardial border was done in short-axis and transverse slices by CMR-experienced investigators (M.F. and N.G.) who interpreted CMR findings blinded to 3DE data.

### Indices of disease severity

Exercise capacity (peak oxygen consumption, VO_2_) and atrial/ventricular tachyarrhythmias documented on electrocardiogram or Holter electrocardiogram monitoring within 2 years from the time of Echo study were used as indices of disease severity as they have been reported to be prognostic for heart failure-related mortality in patients with heart disease.^24^

CPET was performed on a treadmill in 20 rTOF patients, and peak oxygen consumption (VO_2_) was derived. Maximum effort, defined as respiratory exchange ratio > 1.1, was considered a marker of exercise capacity and disease severity. Peak VO_2_ ≤ 16 mL/min/kg suggested moderate impairment in exercise capacity.^25^

Major atrial arrhythmias were atrial fibrillation and supraventricular tachycardia (abrupt salve of three or more consecutive atrial premature beats at a rate of >100 bpm). Ventricular arrhythmias were non-sustained ventricular tachycardia (VT) (>3 beats) or sustained VT lasting more than 30 s (or of any length of time if associated with haemodynamic compromise).

### Statistics

Data were summarized as mean ± standard deviation for continuous variables with normal distribution, median [95% confidence interval (CI)] for other continuous variables, and number of subjects (%) for categorical variables. Echo parameters between groups were assessed by Student’s unpaired *t*-tests. One-way analysis of variance was used to evaluate differences amongst three or more groups. A *P*-value of <0.05 was considered statistically significant. Correlations between variables were evaluated by linear regression analysis. To identify independent factors associated with AAo strain, univariate analysis and multivariate forward stepwise logistic regression analysis were performed. To assess the incremental value of RV 3D-STE + LV 3D-STE + AAo-CS parameters vs. RV 3D-STE indices alone in predicting exercise capacity, *χ*^2^ increase of the multivariate model in logistic regression analysis was determined. Receiver operating characteristic curves were used to quantify the association of arrhythmias with LV conventional and STE parameters. Intra-observer and inter-observer variabilities of Echo parameters were assessed in 15 randomly selected patients. Variabilities were reported in terms of the absolute difference between repeated measurements in per cent of their mean. Reproducibility was assessed by intra-class correlation coefficients, with good agreement being defined as >0.80. Analysis of data was performed using GraphPad Prism (GraphPad Software, LLC, San Diego, CA, USA).

## Results

### Patient recruitment and feasibility

Twenty-five adult patients with rTOF were included in the study. Thirty-two subjects were initially evaluated. Seven patients were ruled out because of coexistent disease (*n* = 5) or poor image quality (*n* = 2). LV 3D-STE, RV 3D-STE, and AAo-CS measurements from all segments were not feasible in a total of 13 subjects (4, 7, and 2 subjects, respectively) due to inadequate myocardial tracking. The overall feasibility of LV 3D-STE was 87% (28/32), the overall feasibility of RV 3D-STE was 78% (25/32), and the overall feasibility of AAo-CS was 93% (30/32).

The baseline age, anthropometric and clinical characteristics, and CPET and CMR data are shown in *Table 1*. Echocardiographic findings are given in *Table 2*.

**Table 1:** Baseline characteristics of patients and controls

	Patients (*n* = 25)	Controls (*n* = 25)	*P*-value
Age at study, years	39.1 (27.4–50.2)	37.6 (24.2–49.8)	0.261
Sex, M/F	15/10	14/11	0.297
Body surface area, m^2^	1.84 ± 0.19	1.92 ± 0.31	0.119
Anatomy			
TOF-PS, no (%)	22 (88)	—	
TOF-PA, no (%)	2 (8)	—	
TOF-AVSD, no (%)	1 (0.4)	—	
Systolic BP, mmHg	122 ± 18	125 ± 15	0.094
Diastolic BP, mmHg	75 ± 10	77 ± 14	0.106
Pulse pressure, mmHg	44 ± 8	46 ± 9	0.323
Age at repair, years	3.8 (1.4–6.3)	—	
Time since repair, years	28.1 (21.4–29.8)	—	
Previous palliative intervention, no (%)	11 (44)	*—*	
Aorto-pulmonary shunt ≥10 years, no (%)	6 (24)		
Mean duration of shunt, years	8.7 ± 2.8		
RVOT reconstruction	10 (40)	*—*	
Follow-up time, years	8.9 (6.5–16.3)		
NYHA functional class			
I, no (%)	18 (72)	*—*	
II, no (%)	7 (28)	*—*	
Sinus rhythm	22 (88)		
QRS duration (ms)	161 ± 19	108 ± 16	0.041
TR			
Mild, no (%)	19 (76)	8 (32)	0.023
Moderate, no (%)	6 (24)	*—*	
RVOT gradient, mmHg	13 ± 7	—	
PR			
Trivial/mild, no (%)	17 (68)	11 (44)	0.019
Moderate or greater, no (%)	8 (32)	*—*	
AR			
Mild	5 (20)	—	
Moderate	4 (16)	—	
CPET^a^			
Peak VO_2_, mL/kg/min	20.7 ± 5.9	23.4 ± 6.5	0.037
Peak VO_2_, % predicted	62.8 ± 7.1	89.1 ± 9.9	0.029
CMR			
RVEDVI, mL/m^2^	135.8 (111.7–158.9)	60.6 (49.4–71.3)	0.0003
RVEF, %	49.2 (43.6–53.5)	57.1 (52.3–59.8)	0.0002
LVEDVI, mL/m^2^	65.6 (51.3–77.5)	60.2 (48.3–70.4)	0.041
LVEF, %	55.4 (49.2–61.5)	63.3 (57.4–68.2)	0.064
Co-morbidities		—	
Coronary artery disease	2 (8)	—	
Dyslipidaemia	3 (12)	—	
Systemic arterial hypertension	1 (4)	—	
Medications		—	
ACE inhibitors/ARBs, no (%)	3 (12)	—	
Beta-blockers, no (%)	4 (16)	—	
Calcium channel blockers, no (%)	1 (4)	—	
Diuretics, no (%)	4 (16)	—	
Anticoagulants, no (%)	2 (8)	—	
Lipid-lowering agents, no (%)	3 (12)	—	

Data are presented as mean ± SD, median (interquartile range), or *n* (%).

ACE, angiotensin-converting enzyme; AR, aortic regurgitation; ARBs, angiotensin II receptor blockers; BP, blood pressure; CMR, cardiac magnetic resonance; CPET, cardiopulmonary exercise testing; NYHA, New York Heart Association; PA, pulmonary artery; PR, pulmonary regurgitation; RVOT, right ventricular outflow tract; TOF-AVSD, TOF with atrioventricular septal defect; TOF-PA, tetralogy of Fallot with pulmonary atresia; TOF-PS, tetralogy of Fallot with pulmonary stenosis; TR, tricuspid regurgitation; VO_2_, peak oxygen consumption.

^a^CPET performed in 20 patients.

**Table 2:** RV, LV, and AAo echocardiographic findings in rTOF patients and controls

Parameters	Controls (*n* = 25)	rTOF (*n* = 25)	*P*-values*
**RV**			
TAPSE (mm)	21.9 ± 5.6	17.8 ± 4.9	0.043
3D-RVEDVI (mL/m^2^)	58.2 (46.7–46.4)	118.1 (97.3–137.9)	0.0001
3D-RVESVI (mL/m^2^)	24.6 (20.2–29.5)	60.8 (51.7–69.6)	0.0003
3D-RVEF (%)	55.1 (48.0–59.9)	46.8 (39.7–51.3)	0.0001
RV-*E_a_* (cm/s)	13.5 ± 2.4	12.6 ± 3.7	0.411
*E*/*E_a_*	4.3 (3.3–5.4)	6.0 (5.5–6.4)	0.068
3D-RVLS (%)	−24.6 ± 2.7	−18.1 ± 2.1	0.004
3D-RVFWLS (%)	−25.8 ± 3.8	−18.4 ± 2.3	0.004
3D-RVFWLS basal (%)	−26.8 ± 3.9	−22.7 ± 3.1	0.035
-RVFWLS apical (%)	−20.9 ± 3.2	−16.8 ± 2.6	0.002
3D-RVAS (%)	−29.6 ± 4.1	−22.9 ± 3.8	0.0003
3D-RVFWAS (%)	−30.2 ± 4.3	−23.1 ± 4.7	0.0003
**LV**			
3D-LVEDVI (mL/m^2^)	59.6 (48.7–69.8)	64.1 (52.4–76.3)	0.065
3D-LVESVI (mL/m^2^)	24.2 (18.1–29.9)	30.9 (23.8–37.4)	0.044
3D-LVEF (%)	62.6 (56.2–67.8)	53.9 (48.3–59.7)	0.069
3D-LV-MI (g/m^2^)	67.8 ± 9.2	93.4 ± 10.3	0.045
LV mass/volume ratio (g/mL)	1.13 ± 0.14	1.24 ± 0.16	0.071
LV-SVI (mL/m^2^)	36.1 ± 7.2	33.6 ± 6.4	0.093
LV-*E*/*A*	1.2 (1.1–1.7)	1.1 (0.5–1.8)	0.086
LV-*E_a_* (cm/s)	12.1 ± 2.2	11.4 ± 2.1	0.057
LV-*E*/*E_a_*	5.5 (3.8–7.2)	8.3 (5.4–10.9)	0.044
3D-LVLS (%)	−21.8 ± 2.8	−18.6 ± 1.4	0.011
3D-LVLS basal-lat (%)	−21.3 ± 3.1	−19.1 ± 2.9	0.043
3D-LVLS apical-lat (%)	−22.5 ± 3.9	−18.9 ± 3.2	0.026
3D-LVLS basal-sept (%)	−18.6 ± 2.7	−17.8 ± 2.2	0.074
3D-LVLS apical-sept (%)	−24.1 ± 4.8	−17.3 ± 4.5	0.012
3D-LVCS (%)	−28.1 ± 4.60	−23.8 ± 5.4	0.023
3D-LVRS (%)	26.3 ± 10.1	25.8 ± 12.3	0.077
3D-LVAS (%)	−38.1 ± 7.9	−30.1 ± 2.6	0.019
Peak LV twist (degrees)	15.7 ± 4.3	13.8 ± 4.2	0.003
Peak BaseRot (degrees)	−6.7 ± 1.8	−6.5 ± 2.1	0.062
Peak ApexRot (degrees)	10.9 ± 3.9	8.2 ± 3.4	0.001
Peak LVtor (degrees/cm)	1.8 ± 0.7	1.5 ± 0.4	0.003
**AAo**			
Sinuses (mm)	28.9 ± 3.6	32.9 ± 5.1	0.027
Sinuses index (mm/m^2^)	15.9 ± 2.7	18.8 ± 3.1	0.019
Sinuses *z*-score	1.4 ± 0.6	2.3 ± 1.2	0.031
STJ (mm)	26.6 ± 2.8	31.8 ± 3.5	0.033
AAo-ED (mm)	27.4 ± 3.9	35.9 ± 4.8	0.023
AAo-ED index (mm/m^2^)	15.1 ± 1.9	20.4 ± 2.5	0.016
AAo *z*-score	1.3 ± 0.7	3.4 ± 1.6	0.015
AAo-SI	2.91 ± 0.83	4.27 ± 0.89	0.029
AAo-D (m^2^/Newton)	75.3 ± 15.4	52.4 ± 13.6	0.035
AAo-Svel (cm/s)	6.5 ± 0.6	5.8 ± 0.5	0.039
AAo-Evel (cm/s)	7.3 ± 0.7	6.9 ± 0.7	0.076
AAo-CS (%)	10.1 ± 2.6	6.7 ± 1.9	0.003
AAo-CS/pulse pressure (%)	16.8 ± 6.3	12.3 ± 4.7	0.003

Data are presented as mean ± SD or median (interquartile range).

3D, three-dimensional; *A*, mitral inflow late diastolic velocity; AAo, ascending aorta; AAo-CS, ascending aorta circumferential strain; AAo-D, ascending aorta distensibility; AAo-ED, ascending aorta end-diastolic diameter; AAo-Evel, ascending aorta wall early diastolic velocity; AAo-SI, ascending aorta stiffness index; AAo-Svel, ascending aorta wall systolic velocity; ApexRot, apical rotation; BaseRot, basal rotation; DT, deceleration time; *E*, mitral inflow early diastolic velocity; *E_a_*, lateral annular early diastolic velocity; LVEDV, LV end-diastolic volume; LVEF, ejection fraction; LVESV, LV end-systolic volume; IVRT, isovolumic relaxation time; LVAS, left ventricular area strain; LVLS, left ventricular longitudinal strain; LV, left ventricular; LVtor, left ventricular torsion; MI, mass index; RV, right ventricle; RVAS, right ventricular global area strain; RVEDV, right ventricular end-diastolic volume; RVEDVI, right ventricular end-diastolic volume index; RVEF, right ventricular ejection fraction; RVESV, right ventricular end-systolic volume; RVFWAS, right ventricular free-wall area strain; RVFWLS, right ventricular free-wall longitudinal strain; RVLS, right ventricular longitudinal strain; sinuses, sinuses of Valsalva; STE, speckle-tracking echocardiography; STJ, sinotubular junction; SVI, stroke volume index; TAPSE, tricuspid annulus systolic excursion; tor, torsion.

### RV-LV data

3D-STE parameters were decreased in rTOF patients compared with controls (*Table 2*). 3D-LVEF was impaired (<50%) in 24% of the patients (*n* = 6). LV twist and 3D-LVAS were impaired compared with controls both in patients with 3D-LVEF < 50% and patients with preserved 3D-LVEF (≥50%) (*Table 3*).

**Table 3:** LV-AAo STE parameters in rTOF patients with preserved vs. impaired LVEF and normal vs. enlarged AAo dimensions

LV parameters						
	rTOF patients (LVEF ≤ 50%)	Normal controls	*P*-value	rTOF patients (LVEF ≥ 50%)	Normal controls	*P*-value
3D-LVLS (%)	−16.7 ± 2.3	−21.5 ± 2.2	<0.05	−17.4 ± 2.6	−21.9 ± 2.4	0.03
3D-LVAS (%)	−28.1 ± 4.1	−39.2 ± 4.3	<0.01	−29.7 ± 3.2	−39.8 ± 4.9	<0.05
LV twist (degrees)	12.9 ± 3.8	15.2 ± 4.1	<0.01	13.9 ± 4.1	15.8 ± 3.9	<0.05

AAo parameters						
	rTOF patients (AAo-ED ≥ 40 mm)	Normal controls		rTOF patients (AAo-ED ≤ 40 mm)	Normal controls	
AAo-CS (%)	6.3 ± 1.7	10.1 ± 2.7	0.002	7.4 ± 1.6	10.2 ± 2.4	0.01

3D, three-dimensional; AAo, ascending aorta; AAo-CS, ascending aorta circumferential strain; AAo-ED, ascending aorta end-diastolic diameter; LVEF, ejection fraction; LVAS, left ventricular area strain; LVLS, left ventricular longitudinal strain; LV, left ventricular; rTOF, repaired tetralogy of Fallot.

Most rTOF patients (84%) had counterclockwise apical rotation and clockwise basal rotation (normal directions), with a prominent reduction in apical rotation and a slight decrease in basal rotation (*Figure 1*). Four patients (16%) had reversed counterclockwise basal rotation and slightly reduced apical rotation.

### Aortic data

Overall, AAo-CS and distensibility were decreased and AAo stiffness was increased in rTOF patients compared with controls (*Table 2*). AAo-CS had a better correlation with AAo diameter (*r* = −0.69, *P* = 0.0001) than AAo stiffness (*r* = 0.49, *P* = 0.01) and AAo distensibility (*r* = −0.53, *P* = 0.002).

The prevalence of aortic dilatation was 24% at the sinuses and 48% at the AAo using an absolute cut-off value of ≥40 mm and 28% at the sinuses and 60% at the AAo using a threshold value of *z*-score ≥ 2.

AAo-CS was decreased compared with controls in both patients with dilated AAo and patients with AAo diameter within normal limits (*Table 3*). Patients with longer duration of pulmonary-to-systemic shunt (more than 10 years) had larger aortic diameter and lower strain than remaining shunt patients (37.8 ± 3.9 mm vs. 32.5 ± 3.6 mm, *P* = 0.03, and 6.2 ± 1.4% vs. 7.3 ± 1.5%, *P* = 0.01, respectively).

### Interventricular and aortic correlations

**Figure 2:**
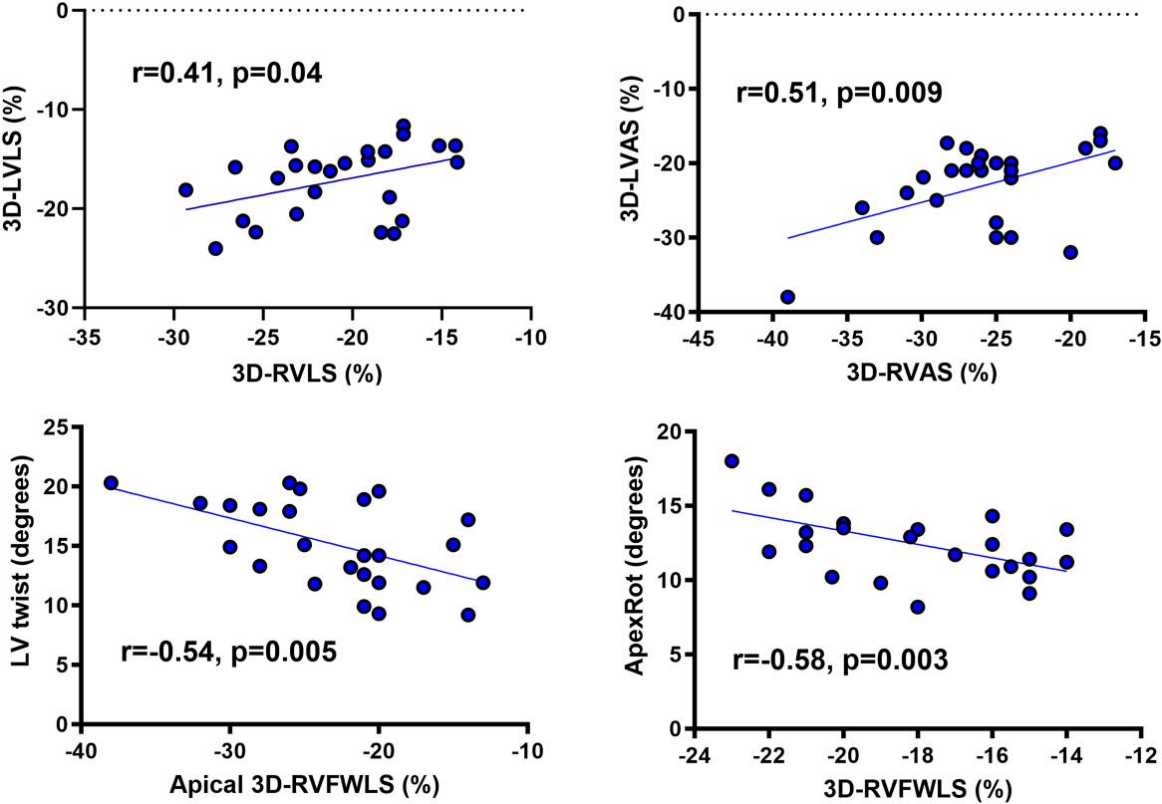
Correlations of LV-RV deformation parameters in rTOF patients. (Top, left) Relation between LV global longitudinal strain (3D-LVLS) and RV global longitudinal strain (3D-RVLS) determined by 3D-STE. (Top, right) Relation between LVAS (3D-LVAS) and RVAS (3D-RVAS) determined by 3D-STE. (Bottom, left) Relation between LV twist and apical RV free-wall strain (apical 3D-RVFWLS) determined by 3D-STE. (Bottom, right) Relation between LV apical rotation (ApexRot) and global RV free-wall strain (3D-RVFWLS) determined by 3D-STE. 3D, three-dimensional; LVAS, left ventricular area strain; LVLS, left ventricular longitudinal strain; RVAS, right ventricular area strain; RVLS, RV global longitudinal strain; RVFWLS, right ventricular free-wall longitudinal strain.

There was a correlation between LV and RV deformation parameters (*Figure 2*) and between LVEF and RVEF determined by CMR and Echo (see Supplementary data online, *Figure S1*). Newsworthy crossed relationships between LV apical rotation and RV 3D-RVFWLS and between RV apical 3D-RVFWLS and LV twist are shown in *Figure 2*.

**Figure 3:**
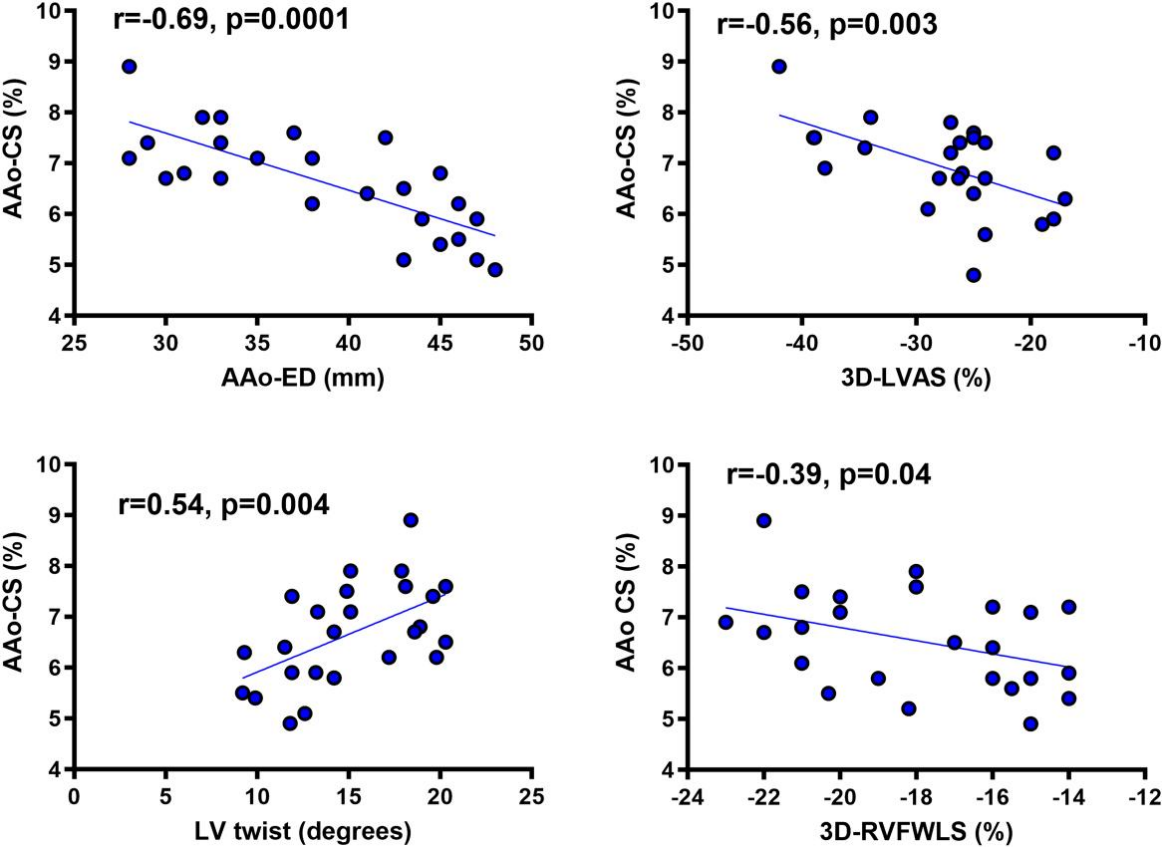
Correlations of AAo-LV-RV deformation parameters in rTOF patients. (Top, left) Relation between AAo-CS and AAo end-diastolic diameter (AAo-ED). (Top, right) Relation between AAo-CS and LVAS determined by 3D-STE (3D-LVAS). (Bottom, left) Relation between AAo-CS and LV twist determined by 3D-STE. (Bottom, right) Relation between AAo-CS and RV free-wall strain (3D-RVFWS) determined by 3D-STE. 3D, three-dimensional; AAo, ascending aorta; AAo-CS, ascending aorta circumferential strain; AAo-ED, ascending aorta end-diastolic diameter; LVAS, left ventricular area strain; RVFWLS, right ventricular free-wall longitudinal strain.

A significant correlation was found between AAo-CS and AAo-CS corrected for pulse pressure with the AAo-SI (*r* = −0.39, *P* = 0.034 and *r* = −0.43, *P* = 0.021, respectively). Both AAo-CS and corrected AAo-CS were reduced compared with controls (*Table 2*). AAo-CS correlated (*Figure 3*) with AAo diameter, LV twist, and 3DE-LVAS. A significant, although weaker, correlation was shown between AAo-CS and 3D-RVEF (*r* = 0.31, *P* = 0.044) and AAo-CS and 3D-RVFWLS (*Figure 3*) and AAo-CS and CMR-RVEF (see Supplementary data online, *Figure S1*). No correlation was shown between reduced AAo-CS and pulmonary artery systolic pressure (*r* = 0.21, *P* = 0.114).

Multivariate analysis (*Table 4*) showed that AAo diameter, 3D-LVLS, 3D-LVAS, 3D-RVFWLS, and 3DRVAS were independently associated with AAo-CS.

**Table 4:** Analysis of ascending aortic wall circumferential strain (AAo-CS) in rTOF patients

Univariate analysis	*r*	95% CI	*P*-value
Age at study	−0.31	−0.27;−3.76	0.041
BSA	−0.34	−0.19;−4.91	0.012
Gender	0.24	0.17;0.63	0.068
NYHA class	−0.16	−0.09;1.23	0.232
SBP	−0.33	−0.21;−0.44	0.015
DBP	−0.26	−0.16;−0.59	0.067
PP	−0.31	−0.26;−0.61	0.043
LVMI	−0.23	−1.08;3.32	0.072
*E_a_*	0.32	0.18;2.74	0.024
*E*/*E_a_*	−0.37	−0.23;−3.76	0.018
AAo-ED	−0.69	−0.49;−2.92	0.0001
AAo-SI	−0.39	−0.18;−0.61	0.034
3DLVEF	0.40	0.21;0.45	0.012
3DLVLS	−0.42	−0.27;−0.44	0.008
3DLVAS	−0.56	−0.31;−0.49	0.003
LV twist	0.54	−0.20;−0.59	0.004
3DRVEF	0.31	−0.24;−0.71	0.044
3D-RVFWLS	−0.39	−0.29;−0.66	0.036
3DRVAS	−0.37	−0.18;−0.57	0.039
Multivariate analysis	β		
AAo-ED	−0.63	−0.42;−0.88	0.001
3DLVLS	−0.42	−0.27;−0.55	0.007
3DLVAS	−0.57	−0.29;−0.71	0.002
3D-RVFWLS	−0.27	−0.22;−0.75	0.041
3DRVAS	−0.32	−0.17;−0.53	0.046

3D, three-dimensional; AAo, ascending aorta; AAo-ED, ascending aorta end-diastolic diameter; AAo-CS, ascending aorta circumferential strain; AAo-SI, ascending aorta stiffness index; β, partial regression coefficient; BSA, Body surface area; CI, confidence interval; DBP, Diastolic blood pressure; *E*, mitral inflow early diastolic velocity; *E_a_*, lateral annular early diastolic velocity; LVAS, left ventricular area strain; LVLS, left ventricular longitudinal strain; LV, left ventricular; LVMI, left ventricular mass index; NYHA, New York Heart Association; PP, Pulse pressure; r, correlation coefficient; RVAS, right ventricular global area strain; RVLS, right ventricular free-wall longitudinal strain; SBP, systolic blood pressure.

### Disease severity

Of the 25 rTOF patients, peak VO_2_ was available in 20 (80%). The peak VO_2_ was 20.7 ± 5.9 mL/kg/min (62.8 ± 7.1% of predicted). Twenty-two patients were in sinus rhythm (88%) at the time of the study, two patients were in junctional rhythm (8%), and one patient was in atrial fibrillation (4%). The following arrhythmias were present in rTOF patients’ history: atrial fibrillation (*n* = 3, 12%), atrial tachycardia (*n* = 2, 8%), non-sustained VT (*n* = 5, 20%), and sustained VT (*n* = 2, 8%). Ten (40%) patients had a history of atrial/ventricular arrhythmias with one or more arrhythmia types. The two patients with sustained VT had no CAD history, negative stress Echo, and mild LV dysfunction, and both patients underwent VT catheter ablation.

There was a moderate correlation between 3DLVAS and peak VO_2_ (*r* = 0.67, *P* < 0.001) and between AAo-CS and peak VO_2_ (*r* = 0.59, *P* < 0.005). A weak relationship was found between LVEF and peak VO_2_ (*r* = 0.34, *P* < 0.05). Analyses for arrhythmia history showed that 3D-LVAS (area under the curve [AUC] 0.796, 95% CI 0.621–0.839), AAo-CS (AUC 0.688, 95% CI 0.547–0.761), and LVEF (AUC 0.603, 95% CI 0.507–0.729) were associated with overall arrhythmia.

**Figure 4:**
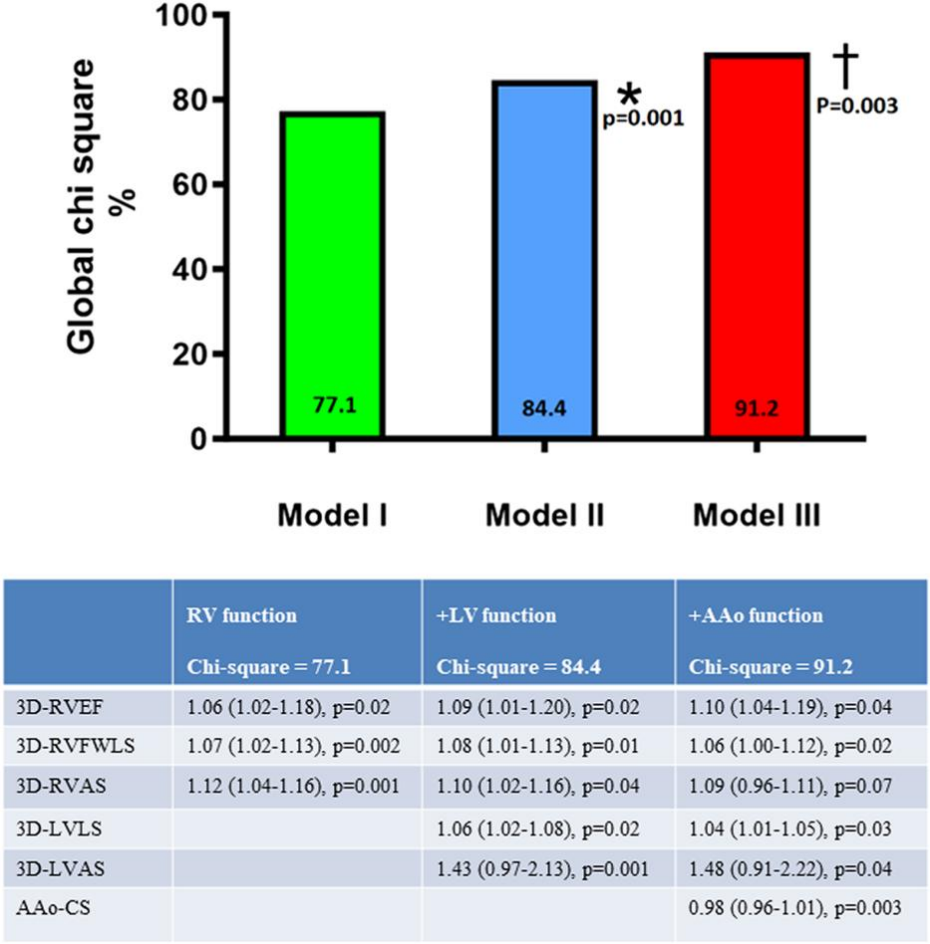
Incremental value of additional LV and AAo dysfunction parameters over standalone RV echocardiographic variables in predicting exercise capacity impairment. Model 1 included RV-STE parameters (3D-RVEF < 48%, 3D-RVFWLS > −22%, and 3DRVAS > −25.5%). Model 2 included RV dysfunction + LV dysfunction parameters (3DLVLS > −20% and 3DLVAS > −30.2%). Model 3 included RV strain + LV strain + AAo dysfunction parameters (AAo-CS < 7.5%). *Model 1 vs. Model 2: *χ*^2^ values 77.1 vs. 84.4, *P* = 0.001; †Model 2 vs. Model 3: *χ*^2^ values 84.4 vs. 91.2, *P* = 0.003. 3D, three-dimensional; LVAS, left ventricular area strain; LVLS, left ventricular longitudinal strain; RVAS, right ventricular global area strain; RVEF, right ventricular ejection fraction; RVAS, right ventricular area strain; RVFWLS, right ventricular free-wall longitudinal strain.

By selecting 3DRVEF < 48%, 3D-RVFWLS > −22%, 3DRVAS > −25.5%, 3DLVAS > −30.2%, and AAo-CS < 7.5% (lower limits of the normal range) as threshold values indicating ventricular or vascular dysfunction, a significant improvement in global *χ*^2^ value was noted with RV-3D-STE + LV 3D-STE + AAo-CS parameters vs. RV 3D-STE indices alone for detecting exercise capacity impairment (peak VO_2_ ≤ 16 mL/min/kg) (*Figure 4*).

### Reproducibility

STE parameter reproducibility was satisfactory (see Supplementary data online, *Table S1*).

## Discussion

This study presents some novel findings in adult patients with rTOF: (i) RV dysfunction was associated with subtle peculiar 3D LV strain/torsion alterations; (ii) AAo strain was decreased even in the presence of normal AAo dimensions and correlated with reduced LV as well as RV 3D-STE parameters; and (iii) LV and AAo changes were associated with disease severity and had an incremental value in detecting exercise capacity impairment.

Our data showed impaired LV deformation and rotational mechanics in rTOF and support and expand previous studies.^26–30^ Area strain measurements are secondary to combined circumferential and longitudinal shortening, and therefore, the more significant reduction in 3D-LVAS achieved in our patients may reflect the combined effect of the reduction of both components. Regarding the distribution of wall motion abnormalities, both LV septal and lateral walls were affected. Septal strain changes are not surprising, as the ventricular septum is common to both chambers, and there is a septal patch resulting from the closure of the ventricular septal defect. LV free-wall abnormalities could be explained by ventriculoventricular interactions because RV can influence LV mechanics by stretching common epicardial fibres shared between both ventricles.

LV twist is an index of LV performance that takes into account the complex helical arrangement of the sub-endocardial/sub-epicardial myofibres. In the present study, LV twist was significantly decreased even in patients with preserved LVEF. This decrease was secondary to a remarkable decrease in LV apical rotation, whereas LV basal rotation was overall slightly reduced or preserved. We observed that online 3D reconstruction showed prominent changes in apical rotation and twist, as depicted in *Figure 1*. Furthermore, we found an interesting significant cross-relationship between RV apical strain and LV twist and between LV apical rotation and RV free-wall strain that corroborates the presence of adverse ventriculoventricular interactions mainly at the apical level. Both decreased apical rotation in adult patients^27^ and reduced torsion due to counterclockwise basal rotation in children^26^ were described, and these different findings could be explained by the different age ranges in these studies. It was shown^31^ that LV impairment begins at the basal level at younger age and expands towards the apex, starting at the endocardium and progressing towards the epicardium over time. In our patients, although a homogenous decrease in strain was observed at different levels of the RV from the base to the apex, deterioration of RV apical strain was better correlated with impaired LV mechanics, indicating a detrimental interaction at this level. Changes in the geometry and strain of the RV apical trabecular component because of volume overload may induce alterations in the apical LV geometry that could lead to decreased apical LV performance. This is consistent with previous studies showing that in patients with rTOF and persistent volume overload, RV cross-sectional area was significantly enlarged at the apical level^32^ and this apical RV dilatation could lead to distortion of apical LV geometry and altered fibre orientation at the cardiac apex. Additional mechanisms trying to explain LV rotational impairment in rTOF patients have been suggested^30^ including congenital or acquired malformations in myofibre orientation, acquired pathological changes at myocardial level such as fibrosis, or abnormal electrical activation. Further research is desirable to better understand the mechanism of LVtor abnormalities in rTOF patients.

Aortic root dilatation is also a well-known feature of unrepaired and repaired TOF. The prevalence of moderate/severe aortic regurgitation varies from 3.5% to 12.5% in patients with rTOF.^33^ The incidence of aortic dilatation defined by various criteria in rTOF patients is reported to be between 14% and 94%.^9–11,23,34^ The pathogenetic mechanisms are believed to be intrinsic genetic-based aortic medial degeneration^9,35^ associated with increased aortic flow attributable to the right-to-left shunt.^11^ Amongst our patients who had previous palliative surgery, those with a longer duration of shunt exhibited larger aortic diameter and reduced strain. However, complete and early repair of TOF with normalized aortic size does not normalize aortic wall stiffness.^12^ Patients with Marfan syndrome, bicuspid aortic valve (BAV), or aortic coarctation also present with aortic dilatation, and in these patients, aortic stiffening precedes dilation, so the former is a potentially useful marker for predicting progressive aortic dilatation.^36,37^ Although aortic medial degeneration is less severe and extensive in rTOF patients than in those with Marfan syndrome, the risk of aortic dilatation is high in patients with histopathological abnormalities. Measuring aortic stiffness and strain as surrogates for aortic medial histopathology may be useful for predicting aortic dilation in rTOF, such as in Marfan syndrome and BAV aortopathy.

We found a prevalence of aortic root and AAo dilatation in rTOF of 28% and 60%, respectively, using a cut-off *z*-score > 2. What the optimal definition of aortic dilatation is controversial. Normal aortic size is related to age, sex, body mass, measurement location, and imaging modality characteristics. Although surgical decision is usually based on absolute aortic diameters, AAo-indexed values and *z*-scores appear to be more accurate for longitudinal follow-up in congenital heart disease.^38^ An observed-to-expected aortic root diameter ratio > 1.5^33^ has been proposed as an alternative definition of aortic root dilatation.

We observed a decrease in AAo-CS and its association with AAo diameter and SI. Aortic strain, especially when corrected for pulse pressure, has been shown to be significantly associated with aortic compliance^22^ and is useful for assessing LV afterload. *In vitro* and *in vivo* validation of arterial ultrasound speckle-tracking algorithm via sonomicrometry has also been reported,^20^ so analysis of vascular strain appears to be a promising tool in evaluating the mechanical properties of the local vessel wall. STE can quantify aortic instantaneous deformation from different directions, is angle independent, is not affected by tethering or translational motion, and, in our patients, had better correlation with AAo diameter than conventional parameters of stiffness and distensibility. It also resulted in early diagnosis since tissue deformation occurs before global structural and functional alterations of the vascular system. Decreased AAo-CS was present even in patients with AAo diameter within normal limits, and this could aid risk stratification of aortopathy and cardiovascular events in rTOF. Furthermore, the accuracy of AAo 2D-STE-derived strain compared with M-mode strain has been described,^23^ with better correlation with the AAo *z*-score.

In our rTOF patients, LV twist and area strain impairment and their significant correlation with AAo strain point up the clinical significance of 3D-STE due to the ability to simultaneously evaluate all LV strain values at the same time from the same 3D data set in complex congenital heart disease. Assessment of impaired ventricular–arterial coupling has a clinical impact on both acquired and congenital heart disease. A correlation between arterial stiffness and LV strain/twist has also been found in healthy subjects^10,39^ suggesting that increased arterial stiffness, which is a common feature of human aging, is associated with a parallel long-term reduction in LV twisting.

A novel finding of the present study was also the significant relationship between AAo strain and RV strain. No correlation was found between AAo deformation and pulmonary artery systolic pressure in our patients, so the association between AAo parameters and decreased LV-RV strain is most likely explained by ventricular geometric interdependence and LV function impairment. RV dysfunction could be both cause and effect of LV dysfunction, worsened by increased aortic stiffness, in a sort of vicious circle. RV dilatation, forced by the relatively non-distensible pericardium, causes LV compression and reduced filling, partly mediated by left septal shift. Progressive RV dysfunction/dilation from increased RV wall stress worsens RV-LV interactions due to decreased RV output and consequently LV preload. LV dysfunction, caused by impaired filling, increased filling pressures, and pulmonary vascular resistance, as well as increased LV afterload, further deteriorates RV function, since the pressure and output developed by the RV depend in significantly on LV function through common myofibre tracts.

Aortic regurgitation was present in nine of our patients, and this may be a further mechanism of biventricular overload in addition to decreased aortic distensibility, as it has been shown both in animals^40^ and in clinical contexts^41^ that aortic valve regurgitation causes volume overload and pathological remodelling of both ventricles. RV remodelling in chronic LV volume overload owing to aortic regurgitation can result from a complex interaction with the remodelled and enlarged LV, beyond the classic, but weak, effect of high pulmonary artery systolic pressure values.

Last, the LV and AAo indices were associated with peak VO_2_ and arrhythmia occurrence and had an incremental value in the detection of exercise capacity impairment. It has been shown that an increase in RV afterload, due to RV outflow tract obstruction and/or pulmonary vascular dysfunction, has a negative impact on exercise capacity in rTOF.^4,24^ In this study, the association between abnormal LV-AAo strain indices and peak VO_2_ is consistent with the negative impact of increased LV afterload on exercise capacity. Likewise, the association between impaired LV-AAo strain indices and arrhythmia occurrence further highlights the role of LV loading conditions and abnormal haemodynamics in the pathogenesis of atrial and ventricular arrhythmias in rTOF.

### Clinical implications

Advanced echocardiography, including 3D assessment of ventricular volumes, is taking on an increasingly important role in the management of patients with rTOF. Since parameters of myocardial deformation are known to better reflect early myocardial dysfunction than volumetric parameters, they could represent a potential guide for treatment in the setting of rTOF. We suggest that STE-derived measures of ventricular strain and torsion should be considered as adjuncts to volumetric parameters of RV and LV function in rTOF patients. Detection of subtle myocardial damage may indicate early beta-blocker therapy to prevent long-term severe LV dysfunction. In rTOF patients with significant pulmonary regurgitation, early detection of RV and LV dysfunctions by myocardial strain and LV rotational mechanics may indicate a need for PVR to reverse RV and LV dysfunctions and improve late clinical outcome.

Furthermore, by evaluating the ascending aorta strain, the results of our study provide new insights into the RV response to chronic volume overload and could help to find additional mechanisms for the deterioration of RV performance and response to therapeutic interventions in the long-term follow-up of patients with rTOF. In these patients, pulmonary arterial stiffness deserves attention because it is related to RV pathologic dilation and can be a target of treatment. With respect to ascending aorta strain, there is currently no consensus on the administration of beta-blockers and angiotensin II receptor blockers to limit progressive aortic root or AAo dilatation. Because replacement of the aortic valve, aortic root or AAo is occasionally required in rTOF, despite the low incidence of aortic dissection in this population, careful follow-up of the proximal ascending aorta after repair is recommended. Aortic surgery should be considered for patients with congenital heart disease and progressive aortic dilatation, particularly in male patients with rTOF and systemic hypertension or patients with rTOF and aortic regurgitation undergoing PVR.

### Limitations

Important technical limitations of 3D-STE are that the speckle-tracking analysis is highly dependent on image quality, and its low frame rate may lead to miscorrelation between frames and affect strain data accuracy. Although single-beat 3D-STE data sets could have been acquired, the image quality of single-beat acquisitions is not currently on an equal level with the image quality of the multi-beat acquisitions.

Furthermore, there is only limited experience cross-comparing intervendor differences in 3D-STE-derived LV-RV deformation measurements. The use of a single vendor is appropriate for early research applications but prevents widespread clinical applications in large populations across multiple imaging platforms and institutions.

As regards the aortic strain, speckle-tracking echocardiographic software developed for myocardial analysis was adapted for vascular strain and optimization of 2D-STE for vascular applications would be desirable.

Finally, this was a retrospective study, exposed to inherent flaws, and included a relatively small number of patients. Normal reference values or outcome data for aortic vascular strain are lacking in large-scale population-based studies. Longitudinal studies could demonstrate the usefulness of LV-RV and AAo strain in predicting major cardiovascular events.

## Conclusions

Speckle-tracking echocardiography reveals subtle LV and AAo dysfunction in adults with rTOF. A correlation is observed between RV and LV strain/torsion changes as well as between AAo strain impairment and LV-RV dysfunction. LV and AAo changes have an incremental value in assessing exercise capacity impairment and should be taken into account, in addition to RV dysfunction, when evaluating post-operative TOF. More extensive studies are needed to investigate the prognostic value of these mechanical parameters.

## Supplementary data

Supplementary data are available at *European Heart Journal – Imaging Methods and Practice* online.

## Consent

Consent to participate: All participants provided written informed consent.

Consent for publication: All authors consent to publication.

## Supplementary Material

qyae015_Supplementary_Data

## Data Availability

The data underlying this article cannot be shared publicly due to ethical restrictions. Anonymized data are available to researchers on reasonable request to the corresponding author.

## Lead author biography

Antonio Vitarelli is an echocardiologist in Rome. He has been working at Toronto, Lund, Charlottesville, Detroit, and Chicago Universities. He was the director of the Echocardiology Operative Unit (clinical and research Unit) from 1992 to 2013 at the Sapienza University Hospital of Rome and is currently Consultant Cardiologist and Adjunct Professor. He is a fellow of the American College of Cardiology and a senior member of various cardiac societies, has written more than 200 articles and a textbook on colour Doppler echocardiography, and is a reviewer for several international journals. His main scientific interests include advanced echocardiography, adult congenital heart disease, valvular heart disease, and cardiac function.
