# Supplementary material for: Biventricular interaction and aortic function in adult patients with repaired tetralogy of Fallot: a two-dimensional–three-dimensional speckle-tracking echocardiographic study
Source: Eur Heart J Imaging Methods Pract. 2024 Mar 2;2(1):qyae015. doi: 10.1093/ehjimp/qyae015 (PMC11195699; doi:10.1093/ehjimp/qyae015)
Supplement: qyae015_Supplementary_Data [file qyae015_Supplementary_Data.zip › Supplementary Table S1.docx]

Supplementary Table S1. Reproducibility of STE parameters

|  | Intraobserver results | | | | Interobserver results | | | |
| --- | --- | --- | --- | --- | --- | --- | --- | --- |
| Variable | Mean difference ± SD | ICC | CV (%) | P value | Mean difference ± SD | ICC | CV (%) | P value |
| 3D-RVLS, % | 7.2±2.4 | 0.86 | 2.59 | 0.002 | 7.9±3.7 | 0.82 | 2.73 | 0.003 |
| 3D-RVFWLS, % | 6.9±2.3 | 0.85 | 2.17 | 0.003 | 7.3±3.1 | 0.83 | 1.89 | 0.004 |
| 3D-RVAS, % | 5.9±2.6 | 0.88 | 3.18 | <0.001 | 6.3±2.5 | 0.84 | 2.88 | 0.001 |
| 3D-RVFWAS | 6.8±2.4 | 0.87 | 2.95 | <0.001 | 7.7±2.4 | 0.83 | 2.47 | 0.002 |
| 3D-LVGLS, % | 6.7±2.5 | 0.95 | 2.94 | <0.001 | 7.5±2.6 | 0.91 | 2.45 | 0.002 |
| 3D-LVGCS, % | 7.9±2.7 | 0.87 | 1.46 | 0.02 | 8.7±2.7 | 0.83 | 1.86 | 0.03 |
| 3D-LVGRS, % | 9.8±4.5 | 0.81 | 2.17 | 0.04 | 10.6±3.4 | 0.79 | 3.59 | 0.05 |
| 3D-LVGAS, % | 5.8±2.6 | 0.95 | 3.14 | <0.001 | 6.3±2.6 | 0.92 | 2.89 | 0.001 |
| 3D-LVTW, ° | 12.1±3.6 | 0.86 | 2.62 | 0.03 | 13.1±3.7 | 0.81 | 2.76 | 0.04 |
| AAo-CS, % | 5.2±2.3 | 0.96 | 3.19 | <0.001 | 6.1±2.9 | 0.94 | 2.91 | 0.001 |

3D= three-dimensional speckle-tracking echocardiography; AAo-CS= ascending aorta circumferential strain; CV= coefficient of variation (calculated as the difference of repeated measurements expressed as a percentage of the mean); ICC= intraclass correlation coefficient for absolute agreement; LVGAS= left ventricular global area strain; LVGCS= left ventricular global circumferential strain; LVGLS= left ventricular global longitudinal strain; LVGRS= left ventricular global radial strain; LVTW= left ventricular twist; RVAS= right ventricular area strain; RVFWAS= right ventricular free-wall area strain; RVFWLS= right ventricular free-wall longitudinal strain; RVLS= right ventricular longitudinal strain; SD= standard deviation.
