# Supplementary figures and images for: Biventricular interaction and aortic function in adult patients with repaired tetralogy of Fallot: a two-dimensional–three-dimensional speckle-tracking echocardiographic study
Source: Eur Heart J Imaging Methods Pract. 2024 Mar 2;2(1):qyae015. doi: 10.1093/ehjimp/qyae015 (PMC11195699; doi:10.1093/ehjimp/qyae015)

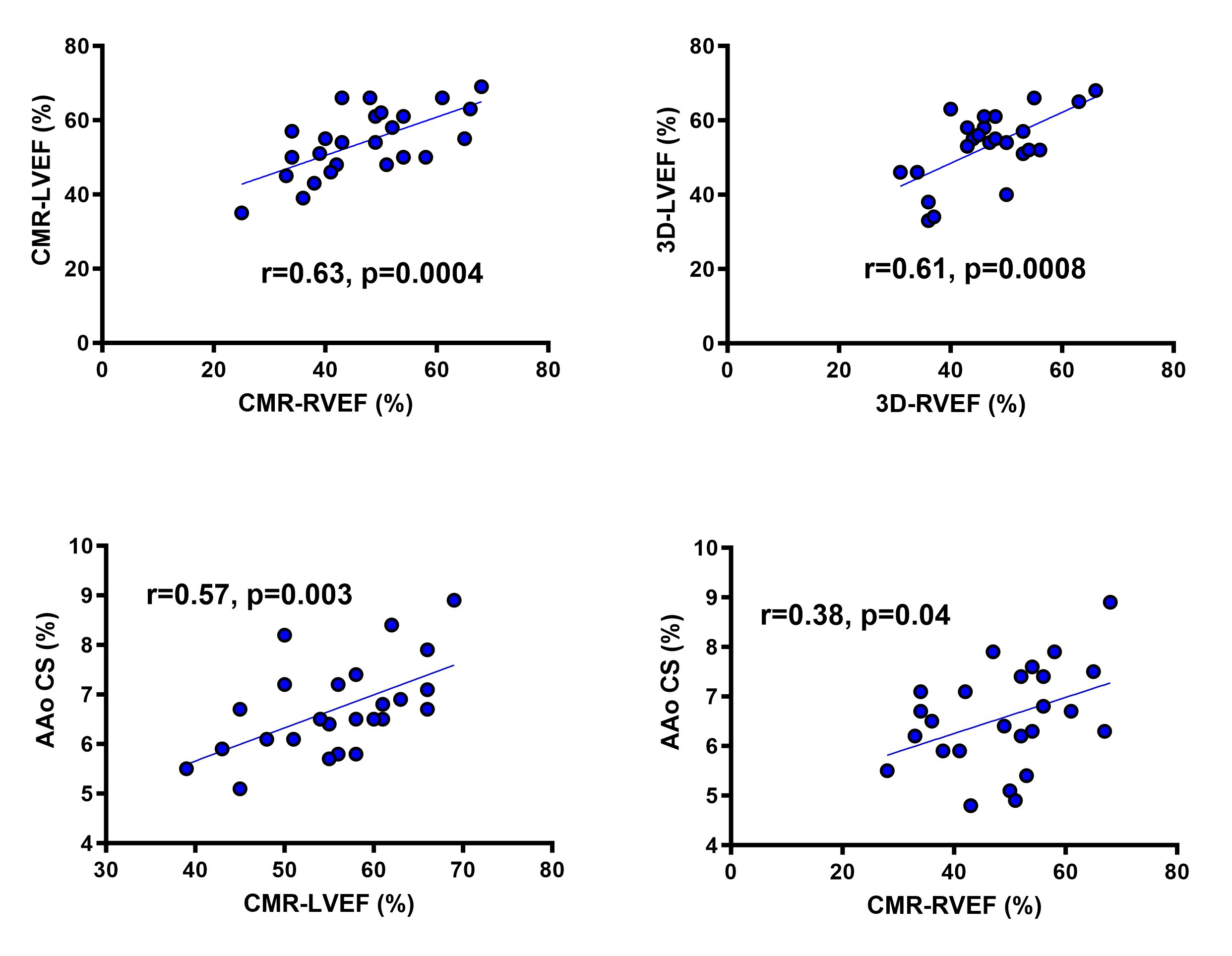

Supplement: qyae015_Supplementary_Data [file qyae015_Supplementary_Data.zip › Supplementary Figure S1 600dpi.jpg]
